# Adolescent alcohol drinking interaction with the gut microbiome: implications for adult alcohol use disorder

**DOI:** 10.3389/adar.2024.11881

**Published:** 2024-01-15

**Authors:** Bruk Getachew, Sheketha R. Hauser, Samia Bennani, Nacer El Kouhen, Youssef Sari, Yousef Tizabi

**Affiliations:** 1 Department of Pharmacology, Howard University College of Medicine, Washington, DC, United States,; 2 Department of Psychiatry, Indiana University School of Medicine, Indianapolis, IN, United States,; 3 Faculty of Medicine and Pharmacy of Casablanca, Hassan II University, Casablanca, Morocco,; 4 Department of Pharmacology and Experimental Therapeutics, College of Pharmacy and Pharmaceutical Sciences, University of Toledo, Toledo, OH, United States

**Keywords:** alcohol use disorder, gut microbiome, adolescence, gut microbiota, toll like receptors

## Abstract

Reciprocal communication between the gut microbiota and the brain, commonly referred to as the “gut-brain-axis” is crucial in maintaining overall physiological homeostasis. Gut microbiota development and brain maturation (neuronal connectivity and plasticity) appear to be synchronized and to follow the same timeline during childhood (immature), adolescence (expansion) and adulthood (completion). It is important to note that the mesolimbic reward circuitry develops early on, whereas the maturation of the inhibitory frontal cortical neurons is delayed. This imbalance can lead to increased acquirement of reward-seeking and risk-taking behaviors during adolescence, and consequently eventuate in heightened risk for substance abuse. Thus, there is high initiation of alcohol drinking in early adolescence that significantly increases the risk of alcohol use disorder (AUD) in adulthood. The underlying causes for heightened AUD risk are not well understood. It is suggested that alcohol-associated gut microbiota impairment during adolescence plays a key role in AUD neurodevelopment in adulthood. Furthermore, alcohol-induced dysregulation of microglia, either directly or indirectly through interaction with gut microbiota, may be a critical neuroinflammatory pathway leading to neurodevelopmental impairments and AUD. In this review article, we highlight the influence of adolescent alcohol drinking on gut microbiota, gut-brain axis and microglia, and eventual manifestation of AUD. Furthermore, novel therapeutic interventions via gut microbiota manipulations are discussed briefly.

## Introduction

Adolescence is a transformative period of human growth bridging developmental chasm between childhood and adulthood. Around the beginning of puberty, critical hormonal, physical, behavioral, and neurodevelopmental changes occur, which culminate through teenage years, and develop further during the mid-20’s [1]. These transformations bring about necessary cognitive and social skills to enable once dependent teens to function as mature and near independent adults [1, 2]. However, some of the consequences of the adolescent neurodevelopmental changes such as impulsivity, risk-taking, sensation-seeking, and novelty-directed behaviors may continue into adulthood. In addition, early maturation of the reward and motivational circuits combined with the protraction of the inhibitory control circuitries, lead to an imbalance between motivational and cognitive-control systems during adolescence. This imbalance can enhance risk-taking behaviors, including substance abuse [2, 3]. Indeed, initiation of alcohol and drug use, often in high doses, are common occurrence during adolescence. Considering the profound neurodevelopmental changes during this time and the ensuing behavioral consequences, adolescence may be considered as a time of both resiliency and vulnerability.

Adolescents often begin consuming alcohol despite their greater susceptibility to its damaging effects [4, 5]. Initiation of alcohol drinking in early adolescence enhances the risk of alcohol use disorder (AUD) in adulthood [5–8]. AUD is a complex brain disorder characterized by an impaired ability to cease or moderate drinking behavior despite adverse effects. Although the exact cause of AUD remains elusive, neurodevelopmental changes, including microglia activity and inflammatory consequences during adolescence, play a pivotal role [9, 10]. As neuronal maturation and refinement peak during adolescence, the process of pruning, entailing removal of weak synaptic connectivity and enhancement of myelination continue into adulthood [11]. Thus, there is a reduction of gray matter and an increase in the white matter volume [12–14]. This is accompanied by enhanced connectivity, which allows faster speed and efficiency of information flow across relatively distant regions [15–17]. Two distinct and notable circuits in this regard, are the mesolimbic reward pathway and the prefrontal cortex inhibitory circuit (PFCX), both of which are critically involved in the complex social and cognitive processes [18, 19]. The mesolimbic dopamine (DA) circuit, however, as mentioned earlier, matures early in adolescence, whereas there is a delay in PFCX development, resulting in a vulnerable window.

In this review, we focus on the neurodevelopmental stages of adolescence, including role of key players such as gut microbiota and microglia and the influence of alcohol use on these parameters. Moreover, potential exploitation of such components for therapeutic purposes are elaborated on.

## Adolescent neurodevelopment

The term “adolescent” describes a young person in the process of developing from a child into an adult [20]. Adolescent neurodevelopment is conserved throughout evolution and across species, signifying its crucial importance in acquiring necessary behavioral skills for transitioning into adulthood [1, 3]. These skills include attaining heightened reward sensitivity, acquiring peer-directed social interaction, and cognitive enhancement, all of which are essential in achieving maturity [2, 3, 21, 22]. Heightened reward sensitivity is considered a milestone necessary to facilitate approach toward novel stimuli and learning from new surroundings and social interactions [23]. However, risk-taking, novelty-seeking, and sensation-seeking behaviors, may predispose adolescents to alcohol and drug use [24, 25]. Curiously, these behaviors may also be manifested in animals [26, 27], suggesting that certain neurobehavioral characteristics of adolescence may have biological causes.

### Neuronal refinement during development

Neuronal refinement and maturation continue throughout adolescence, as at birth and even during adolescence, there are more neurons (about 4–5 times) than in adulthood [28–30]. Approximately 50% of the synaptic connections in selective regions are lost due to synaptic pruning [31], which is believed to ensure establishment of appropriate connectivity [31, 32], reduction in energy use, and increase brain efficiency [33, 34]. This process is also affected by myelination that begins early in life, peaks through adolescence and continues into adulthood [11]. Thus, increase in myelination and decline in synaptic connection help refine brain connectivity into the adult form [35]. However, myelination can be impacted by neurotoxic agents such as alcohol, which can poise great danger to the maturing brain.

Some of the adolescent synaptic pruning appears to be experience dependent [35]. For example, heightened stress exposure and alcohol consumption during adolescence, can affect neurodevelopmental resiliency [36]. On the other hand, enriched environment during adolescence can induce a variety of beneficial changes in the expression of genes in critical brain areas such as in striatum, an area that plays a pivotal role in motor and motivational behaviors [37]. Myelination is also experience-dependent as it helps stabilize axonal pathways [38]. It is believed that myelination, in concert with synaptic pruning help with the “rewiring” of brain, particularly the prefrontal cortex (PFCX), which is critical for many adult-type behaviors including cognitive functions [39].

### Reward and impulsivity during brain development

DA system, essential for detecting, responding to, learning from reward, cognitive control, decision making and motivation [40, 41], undergoes significant transformation during adolescence [42]. Specifically, there is a loss of up to 50% of DA (D1) receptors in some areas, a compromised clearance of which, results in a reduction in social play and social exploration [43]. However, in other areas, DA activity may increase two-to seven-fold during adolescence [44, 45]. Thus, the mesolimbic DA pathway, considered to be the reward circuitry, is maximally developed in adolescence [18, 46, 47], which corresponds to peak in reward-seeking in mid-adolescence (i.e., approximately 14–15 years) that gradually declines into adulthood [25, 48, 49]. On the other hand, PFCX DA system, considered to be critical in inhibitory control of risk taking, has a protracted maturation [18, 50, 51]. This protraction results in developmental immaturities in cognitive control, attentional regulation, and response inhibition of behaviors [2], and may contribute to the persistence of certain maladaptive behaviors such as alcohol and drug use in adolescence [52, 53].

Dysregulation of PFCX behavioral control systems is associated with impulsivity, which contributes to alcohol seeking and use during adolescence, particularly, in stressful and arousing situations [54, 55]. This impulsivity may even continue into adulthood binge drinking (aged 18–30) [56]. Animal models of AUD also show impulsivity and risky choice behavior if PFCX is dysregulated, suggesting biological basis for such behavior [57, 58]. Excess alcohol use, in turn, by damaging neuronal cells, could lead to dysregulation in PFCX, further exacerbating aberrant behaviors (impulsivity/drug seeking) which can lead to drug addiction. Therefore, adolescent alcohol consumption can be considered a risk factor in AUD development in adulthood [59, 60]. Thus, delaying the onset of alcohol drinking, during this period of vulnerability, can significantly reduce the risk of AUD [2].

### Environmental and non-neuronal factors during development

Adolescent engagement in risky behaviors commonly occurs in social situations [61–63]. Shaping and refinement of the brain neuronal system during this period is also impacted by exposures to environmental factors. Microbiomes, discussed in detail below, have recently attracted considerable attention as an important influencer of the brain function and affected by environmental factors such as diet, chemicals, etc. Interestingly, it was suggested that early life antibiotic-induced microbiota disruption may have subtle but enduring effects on the brain function and social behaviors [64].

Microglia, also discussed below in detail, are non-neuronal cells that constitute only 10% of the total CNS cells [65]. Nonetheless, they perform important task of surveying the environment and responding to insult [66, 67]. Microglia are considered CNS phagocytes, which also undergo significant changes during adolescence [68]. These changes contribute to neurodevelopmental fine-tuning [69–71]. Such as increase in brain efficiency, and synaptic pruning throughout cortical and limbic structures [71–73]. Moreover, by influencing early myelin formation and removing aberrant myelin [74], myelination is optimized. Interestingly, microglia may also play a role in dopaminergic circuits refinement which, as discussed above, are critical in reward-seeking and social behavior [75].

## Microbiome and neurodevelopment

The gut microbiome (GM) is an ecosystem of 100 trillion commensal microbes, complex in composition and abundance, that mainly colonize the gut [76, 77]. Although the terms microbiota and microbiome are often used interchangeably, microbiota refers to the actual microbes, whereas microbiome refers to the microbes and their genes. The colonization of the gut starts at neonatal period and continues throughout life. During infancy, the ecosystem is unstable, but GM develops into a highly diverse and robust community in adulthood [78]. It was thought that the collective genome of microbiota, the microbiome, encodes 100 times more genes than the human genome [79]. However, recent in-depth analyses suggest only a slightly higher number of microbiomes compared to the human genome [80–83]. GM is essential for the maintenance of the host’s health including innate and adaptive immune system [80], food digestion, fermentation of otherwise indigestible carbohydrates and fibers, energy production, synthesis of several vitamins (e.g., vitamins K and B) and the metabolism of bile acids, sterols, and xenobiotics [81, 82]. GM can produce or release neurotransmitters, choline and its metabolites as well as short chain fatty acids (SCFAs). These products are secreted into the gut lumen, transported across the epithelial barrier, and carried to the effector organs including the brain, via the bloodstream. The gut microbiome, due to its immense impact on human equilibrium, immune function, neurology, mental health, and aging process, is now commonly referred to as a new metabolic “organ” [80–83].

Maturation of GM is critical for neuronal maturation and brain development [83]. Many studies show GM maturation parallels the temporal course of brain development. Using several experimental approaches, including germ-free (GF) animals, and antibiotics, host microbiota’s effect on CNS functions have been studied [84–86]. For example, some antibiotics such as minocycline have profound acute effect on the microbiota diversity and composition [87, 88]. Moreover, the fact that most critical development of host immunity occurs within the first few years of life, which coincides with the maturation of the GM, reinforces the notion that GM is also involved in immune system development [89, 90].

The synchronized communication between the CNS and GM via GBA is critical in shaping the neurodevelopment and influences brain’s biology under homeostatic conditions [91, 92]. Some of these functions include regulation of the permeability of BBB [93–95], and glial functions [91]. GM’s metabolic products SCFAs, vagus nerve, and microbe-associated molecular patterns (MAMPs) (such as Toll-like receptors (TRLs) are the mechanisms purported to facilitate communication between GM and CNS [96]. Most TLRs, a family of pattern-recognition receptors that enable the recognition of conserved structural motifs of wide array of pathogens that drive inflammation, are expressed in the CNS, mainly in glial cells [97]. SCFAs monitor and integrate gut functions with emotional and cognitive centers of the brain. SCFA also regulate peripheral intestinal functions, intestinal permeability, and immune activation [98]. Indeed, microglia from GF-mice display a range of abnormalities that are dependent on GM SCFA. A specific pathogen-free (SPF) mice constitutively lacking the SCFA receptor FFAR2 displayed a similar aberrant phenotype to GF animals [99], suggesting that GM metabolite, SCFAs, and microglia are involved in the bidirectional crosstalk between GM and the brain.

It is not surprising, therefore, that dysbiosis or disruption of intestinal microbiota homeostasis can lead to variety of diseases [100], including cardiovascular [86], inflammatory bowel disease [101], and type 1 and type 2 diabetes [102, 103]. Common also, are CNS disorders such as anxiety, depression and substance abuse [82, 104, 105]. Dysbiosis can be caused by environmental factors including diet [106], disruption of circadian rhythms [107], and alcohol consumption [107], where the latter is discussed in more detail below.

## Microglia and neurodevelopment

Microglia, considered the immune cells of CNS, are primarily responsible for neuroimmune responses and neuronal development [108, 109]. They facilitate the maturation and survival of neuronal progenitors and proper network integration during CNS development [110]. In general, there are three phases: early, pre- and adult microglia. Microglial maturation phases are defined by expression of a subset of genes corresponding to the core set of microglia functions [111]. Therefore, microglia show heterogeneous transcriptional profiles in the embryonic, early postnatal, and adult, depending on their microenvironment in CNS [112–114]. Early on, before BBB development, microglia derive from immature erythromyeloid progenitors, and migrate from the yolk sack blood islands to CNS [111, 115]. During late gestation and early postnatal development, embryonic microglia proliferate and colonize the whole CNS [111]. A few weeks after birth, microglia transition to “adult microglia” stage, in which they constantly survey their immediate surroundings and actively maintain homeostatic conditions by phagocytizing neuronal debris [116], and interacting with neighboring CNS cells [117]. They achieve these through the dynamic extension and retraction of their processes [118, 119].

Microglia can assume different phenotypes and retain the capability to shift functions to maintain tissue homeostasis depending on the influence of stimuli from the environment [120]. For example, during infection or injury, microglia switch from a homeostatic surveillance state to an activated state to facilitate antimicrobial or tissue repair to restore homeostasis [108]. Importantly, microglia can either be stimulated by GM toxin lipopolysaccharide (LPS) to a pro-inflammatory (M1) phenotype where they would express pro-inflammatory cytokines, or by IL-4/IL-13 to an anti-inflammatory (M2) phenotype for resolution of inflammation and tissue repair [120]. Given their dual role in immune and developmental functions, it may be expected that microglial dysregulation would contribute to neurodevelopmental disorders. Indeed, microglia overactivation could lead to neuronal damage and onset/progression of several neurodegenerative and neurodevelopmental disorders [121]. In addition to pro-inflammatory cytokines, other bioactive substances released from overactivated microglia, such as ROS and glutamate could also play a role in microglia-dependent neuroinflammation [122], and/or neuronal loss [123, 124].

Since microglia can also shape neurodevelopmental fine-tuning and complex neurodevelopmental programing [125], their transient reduction at critical stages of development can alters synaptic plasticity [126]. Microglia interaction with various cellular components including neuronal activity and synaptic formation, leads to establishment of novel functional neural network. Thus, microglia by inducing synapse formation during development, monitor the functional state of synapses in adulthood [70]. Moreover, during early brain development, microglia’s main functions include synaptic remodeling, regulating the number of neurons through mechanisms of programmed cell death (apoptosis) [71, 125], and shaping of the neuronal circuitry. Thus, early on in life, the brain being highly plastic, contains excess number of immature synaptic connections and is shaped by sensory experience [70]. The over-crowding of neurons is subsequently “pruned,” or eliminated, primarily via microglia, to allow functional connectivity during development [126, 127]. In addition, microglia’s involvement in myelination (via oligodendrogenesis), during development and throughout life, allows efficient and critical neuronal communication [74]. Curiously, recent evidence implicates microglia’s own programmed cell death via pyroptosis, autophagy, and ferroptosis in neurodegenerative diseases, including Alzheimer’s disease (AD) [128].

Microglial activity, governed in part by cytokines, chemokines, neurotransmitters, and other signaling molecules [129], is highly sensitive to environmental cues. As such, GM has emerged as a central player in microglial maturation and activation [120]. Sophisticated crosstalk between the CNS and the gut microbiome, and critical interdependency between microglia and GM, where the latter facilitates microglia’s development, are now well-established [130]. However, the exact mechanisms of such communications are not well understood. Below, our current knowledge of GM-microglia interactions in relation to brain maturity and adolescent drinking are reviewed.

## Microbiota-microglia interaction and neurodevelopment

That GBA plays a pivotal role in regulating microglial maturation and function during critical windows of development is well recognized [131, 132]. Microglia, in turn, is one of the key cellular intermediates linking CNS with GM. Distinct developmental stages are present during which there is heightened microglia susceptibility to immune mediators and environmental cues [110, 113]. For example, environmental exposure to chemicals such as alcohol can disrupt microglia development and maturation, primarily due to dramatic changes in microbiota. Similarly, antibiotics-induced loss of GM causes microglia to assume an immature status reminiscent of developing juvenile microglia [133]. On the other hand, recolonization of the gut with complex microbiota restores its plasticity, a finding that was also confirmed in mice born from GF maternal mice [134]. Indeed, GF-mice exhibit a wide range of microglia abnormalities including increased density and distribution across various brain regions and altered cytometric expression patterns for developmentally regulated proteins [99]. Moreover, such microglia are less reactive when challenged with LPS, again, suggesting GM’s crucial role in microglia maturation and neuronal function [99]. Interestingly, microglial changes appear to be dependent on SCFAs, as specific pathogen-free (SPF) mice constitutively lacking the SCFAs receptor FFAR2 display a similar aberrant phenotype as seen in GF animals [99]. Furthermore, GF and antibiotic treatment not only disrupt typical microglial spatial network throughout the brain but also result in forming atypical contacts between processes of adjacent cells [99].

Importantly, however, is the finding that even transient perturbations in microglial function could have life-long effects on neuronal patterning, functional connectivity and behavior [135, 136]. Thus, it has been demonstrated that a transient reduction in microglia number at critical stages of development alters synaptic plasticity including differentiation and maturation of precursors into neurons or neurogenesis [126, 137]. This is because most newborn neurons undergo apoptosis and are phagocytosed by microglia as part of normal neurodevelopment. However, over time, this process becomes limited to neurogenic niches of the adult brain [127]. In addition, microglia not only play a critical role in debris clearance but may also facilitate neuroblast differentiation in response to signals [138]. Maternal immune activation results in accelerated microglial maturation that exhibit adult microglia phenotype [110, 113] and can present with detrimental consequences including neurological disorders that continue long after the microglia phenotype is restored [113]. On the other hand, microglia’s expression of pro-inflammatory cytokines such as TNF- α, IL-1 β, and IL-6 and trophic factors, help mediate the interactions between the host’s microbiome and the developing brain [139], resulting in refinement of functional neuronal circuits [131, 132, 140]. It was demonstrated recently that microglia’s secreted factors directly increase differentiation of human neural stem cells to a dopaminergic lineage [83]. However, whether microglia are involved in heightened reward-seeking and/or risk-taking including development of AUD, remains to be determined.

Collectively, these findings suggest that bidirectional crosstalk between the gut and the brain may influence disease pathogenesis. Thus, alteration in GM during the early stages of development may have long-lasting effects on the GM composition throughout the lifespan with clear implications for the immune system as well as neuronal development. Because excessive alcohol consumption results in dysbiosis and microglia alteration, it is not surprising that AUD would be associated with neurological diseases [141]. Below, further association between alcohol, GM, and microglia in relation to neurodegenerative/neuropsychiatric disorders is elaborated.

## Adolescent alcohol drinking

Alcohol is the most used drug among adolescents [142–144], where experimentation and initiation usually begins in early adolescence (50%–70% of 15 year-old use alcohol) [145], and peaks during young adulthood (18–24 years of age), where binge drinking is more common [146]. US prevalence of binge drinking in adolescents aged 12–17 and 18–25 are 4.7% and 34.9% respectively [147]. Binge drinking is considered consuming 4 or more drinks for females and 5 or more drinks for males within 2 h [143, 148]. A single drink consists of about 14 g of pure alcohol, which is found in 12 ounces (355 mL) of regular beer (usually containing 5% alcohol); 5 ounces of wine (usually containing about 12% alcohol); or 1.5 ounces of distilled spirits, which is about 40% alcohol. Although, binge drinkers drink less frequently, they drink more alcohol per drinking episode achieving a blood alcohol level (BAL) topping 0.08% (>80 mg/dL) and hence increasing alcohol-associated risks and consequences [143, 148, 149]. A small percentage (10%) of binge drinkers are considered heavy binge drinkers, where 10 or more drinks are consumed per occasion, and yet 5% are extreme binge drinkers where over 15 drinks is consumed in a binge session [142, 150]. Epidemiological report indicates that early initiation of alcohol drinking before the age of 15 years increases the risk of AUD in adulthood by fourfold [6, 7]. About 30%–40% of adolescent binge drinkers, i.e., 1.6% of 12–17 year-olds and ~14% of older adolescents, meet criteria for AUD [147, 151, 152]. Although males are overrepresented in the extreme binge drinkers, the gender gap is narrowing [142, 153].

In addition to adolescent drinking, individuals with fetal alcohol syndrome disorder (FASD), a heterogeneous group of conditions defined as the physical, behavioral, and learning impairments that occur in the offspring of women who drank alcohol during pregnancy, may also exhibit increased risk of substance abuse including AUD in adulthood. Thus, alcohol exposure may impact behavioral outcomes throughout neurodevelopmental period where the earlier the exposure, the worse the outcome [154]. However, disentangling underling factors in each case remains a challenge [155].

It is noteworthy that adolescents, compared to adults, are insensitive to various intoxicating effects of alcohol such as motor incoordination, social impairment, and sedation [3]. It is thought that adolescent-typical insensitivities to aversive stimuli in the presence of greater reward sensitivity contribute to their proclivity to associate more benefit and less cost to alcohol and drug use. This could encourage pursuit of or continued engagement in risky activities, particularly when prior activities proved rewarding but without disastrous consequences [62, 63, 156].

## Alcohol use disorder and microbiome

A potential connection between GM and AUD was suspected since mid 1980s. Initially, the role of GM in alcoholic liver disease was intensely investigated. Later, possible role of GM in addiction to alcohol was advocated. With our advancement in understanding of the GBA, it is anticipated that novel GM-targeted therapies will become available [157].

It is important to reiterate that harmful consumption of alcohol (alcoholism) is responsible for approximately 5.3% annual deaths in all age groups, and at an alarming rate of 13.5% for the younger age group of 20–39 years old [144]. Although alcoholism has been studied for decades, only relatively recently the examination of gastrointestinal (GI) microbiome and its impact on AUD has been intensely investigated. An initial observation reported that the content of Gram-negative anaerobic bacteria in jejunal aspirates from alcoholic individuals were significantly higher compared to control individuals [158]. Animal studies, confirmed this involvement where it was shown that more than 10 weeks of ethanol ingestion in rats led to significant dysbiosis of the colonic microbiome [159]. In subsequent years, many sequencing studies of the microbiome from rodent models of alcoholism, humans with AUD, as well as non-human primate studies of addiction have solidified GBA’s importance in alcohol addiction [157].

Thus, GM not only plays an important role in development of AUD but also in a variety of neurological and neuropsychiatric diseases including Parkinson’s disease, Alzheimer’s disease, depression, and autism spectrum disorder [160–162]. Chronic alcohol consumption can cause changes in the composition of GM and impair the gut mucosal barrier as well as homeostasis. Once the mucosal barrier is compromised, LPS from GM is released and translocated to peripheral blood circulation, where it acts on TLR4 [163]. Activation of TLR4 can lead to increases in proinflammatory cytokines which further disrupt BBB and hence result in further neuroinflammation [164], a major contributor to AUD. For these reasons it has been suggested that the gut–brain axis might be a potential target to reduce alcoholic relapse risk.

In addition to the central effects of AUD, GM dysbiosis, can lead to liver disease. Indeed, GM changes occur in parallel to liver injury, with an increase in endotoxin-producing bacterial taxa, leading to cirrhosis and alcoholic hepatitis. In this regard, AUD effect on GBA can further potentiate alcohol misuse and hasten hepatic encephalopathy. Thus, strategies that address both alcohol cessation and microbiota alteration are needed for meaningful improvement in all AUD spectrum [165].

Furthermore, a plethora of indirect evidence point at the involvement of GM dysbiosis in microglia activation (discussed below) and AUD. For example, GM metabolite SCFAs can cross BBB and affect microglia directly [166]. Both infiltrating macrophages and microglia become activated in response to tissue damage and can release proinflammatory cytokines, which may contribute to neuroinflammation and BBB breakdown [167, 168]. Orally administrated mixture of the three major SCFAs acetate, propionate and butyrate can sufficiently drive maturation of microglia [169]. Of these, butyrate has been demonstrated to possess multiple benefits, including enhancing the gut barrier, reshaping the gut microenvironment, and repressing the inflammatory progression. Moreover, butyrate has shown to be neuroprotective against alcohol toxicity in an *in-vitro* model [170].

Alcohol abuse via changes in GM composition and metabolic function can lead to oxidative stress and leaky gut (allowing bacterial passage into the lumina), and subsequent development of alcohol-related diseases [81, 171]. Also, GM dysbiosis by disrupting microglial maturation and activation can causes behavioral changes associated with AUD. However, despite frequent reports of dysbiosis in AUD patients, microbiome-targeting therapies for this disorder awaits clinical trials (see also below for more detail).

## Alcohol use disorder and microglia, and role of toll-like receptors

Microglia involvement in AUD pathology is amply supported by the findings that prolonged and heavy exposure to alcohol can not only lead to appreciable reduction in glial cell numbers in both temporal and frontal cortices [172], but also to impairment of neuronal and glial cell functionality [173]. In the developing brain, these effects are more pronounced and extend to cerebral white matter, corticolimbic system and cerebellum (especially the vermis) [173]. Cortical microglia, however, show remarkable morphological plasticity as they rapidly deactivate following acute severe alcohol exposure [174]. Following chronic high alcohol exposure, there is a marked increase in microglia activation [167, 175], accompanied by high levels of proinflammatory mediators and reactive oxygen species that can lead to tissue damage and cell death [103]. Conversely, chemical depletion of microglia, can block the production of inflammatory mediators in the brains of mice after acute binge ethanol withdrawal [176].

Epidemiological studies, based on FASD, also suggest a role for microglia in early neurodevelopment [177], as areas that are dependent on neuroglial cells for their formation such as corpus callosum and anterior commissure exhibit abnormal glial migration [178] and underdevelopment [179]. Moreover, during brain growth spurt, characterized by rapid glial cell proliferation and maturation, ethanol exposure can lead to microencephaly, suggesting potential effect of ethanol on proliferation, growth, and maturation of glia [180]. Likewise, during adolescence, binge drinking causes devastating effects as reflected in morphological changes in hippocampal microglia that can last over 1 month [181]. Accompanied neuroinflammatory processes induce behavioral changes such as sedation and alcohol withdrawal symptoms including memory impairment, neuronal cell death and diminished neurogenesis [182, 183]. Insensitivity to sedative effects to alcohol, blackouts and kindling, contribute to exacerbation of withdrawal episodes with each cycle of withdrawal during adolescence [184, 185].

Chronic alcohol consumption induces microglia proliferation [167, 186, 187] and microglia morphological changes reflective of a proinflammatory phenotype in a context-dependent manner [9, 186]. During context-dependent activation of microglia, prior insults are recalled, resulting in amplified responses to a second inflammatory insult [188, 189]. This suggests that prior ethanol exposure potentiates a subsequent microglia response that is primed by initial alcohol exposure. Alcohol can directly activate microglia to increase expression of proinflammatory chemokines and cytokines. The chemokines and cytokines in return, can alter sensitivity to alcohol-induced sedation, alcohol withdrawal severity [182], memory impairment [183], as well as alcohol drinking patterns [190].

Alcohol-enhanced microglia-specific immune responses can be blocked by minocycline, a microglia activation inhibitor [191]. This blockade of microglia immune response alters alcohol-induced motor impairment decreases alcohol self-administration in mice [192], and attenuates withdrawal-induced anxiety and relapse drinking in rats, suggesting that microglia may be the critical mediator of alcohol behavioral effects [193]. Minocycline also reduces traumatic brain injury (TBI) induced by microglial activation [194]. Since alcohol use is associated with microglial activation, it would be reasonable to expect that adolescent binge drinking may enhance TBI. However, the effects of adolescent binge drinking on microglia and potential use of minocycline in AUD remains to be investigated.

Adolescent alcohol drinking impacts central inflammatory cells and signaling molecules [167]. Sensitized microglia can interfere with homeostasis by decreasing expression of homeostatic genes [195]. For example, several genes in Toll-like receptor (TLR) signaling pathways are activated by alcohol [96]. TLRs are important mediators of inflammatory pathways in the gut and play a crucial role in maintaining the balance between commensal bacteria in the gut and the mucosal immune system [196]. TLRs are evolutionarily conserved receptors belonging to the family of pattern recognition receptors (PRRs) which play a vital role in immune responses. Indeed, TLRs hold a key position in the first line of defense against pathogens because of their ability to recognize the conserved pathogen-associated molecular patterns (PAMPs) that are conserved structures of the pathogens. Activation of PRRs results in the downstream transcriptional activation and expression of numerous inflammatory mediators. In addition, PRR signaling also leads to the triggering of various processes involved in autophagy, cell death, cytokine processing, and phagocytosis. Thus, TLRs are directly involved in the regulation of inflammatory reactions and activation of the innate or adaptive immune responses for the elimination of infectious pathogens and cancer debris [196].

To date, 222 TLRs have been identified in invertebrates and 28 TLRs in vertebrates. Depending upon their functionality and location in the host cell, TLRs are further categorized into two types: 1. Cell membrane TLRs, which are expressed in their active form on the cellular surface. They include TLR1, 2, 4, 5, 6, and 10.2. Intracellular TLRs, which are expressed within the host cells on the organelle biomembranes like endoplasmic reticulum (ER), endosomes, and lysosomes. They include TLR3, 7, 8, and 9 [196]. TLR4 is the major pattern recognition receptor of bacterial endotoxin, LPS [163]. Although endotoxins are not generally believed to cross BBB [197], they can induce proinflammatory microglia. Indeed, in TLR4 knockout and postmortem tissue of AUD patients, there is breakdown of BBB [198]. Interestingly most of the TLRs are expressed in microglia and astrocytes [164, 199, 200]. n addition to microglia, peripheral macrophages can be recruited into the CNS under pathologic conditions and may serve to amplify ongoing neuroinflammation [201]. Alcohol’s activation of TLRs triggers downstream stimulation of nuclear factor-κB (NFκB) and the induction of genes that encode inflammation-associated molecules such as cytokines [202, 203]. Thus, activation of the TLRs can significantly contribute to neuroinflammation [204]. Indeed, increased TLR4 activation is often the reason for neurodegeneration exacerbation [205]. Hence, it may be concluded that at least some of neurodegenerative consequences of heavy alcohol drinking might be mediated via TLR4 stimulation.

As mentioned earlier, adolescent exposure to alcohol significantly increases the risk of AUD in adulthood. Although the reason(s) behind this association is not fully known [206], it may be speculated that alcohol’s priming effect of microglia or changes in TLRs may have major roles. Interestingly, TRLs are also involved in bidirectional communication between GM and CNS and are believed to play an essential role in regulating intestinal barrier permeability and maintaining intestinal microbial homeostasis. The intestinal microbiota, in turn, plays an essential role in TLR ligand activation and distribution [207]. Thus, alcohol-induced dysbiosis in adolescence may be a major contributory factor to AUD development in adulthood. This discovery, as discussed below, may present with novel interventions in AUD.

## Possible microbiome directed therapies against alcohol use disorder

Based on above discussion, it is likely that manipulations of GM may offer a novel intervention in AUD. In this regards, fecal microbiota transplantation (FMT) in patients with alcoholic liver disease [208, 209]. and more recently for the treatment of AUD in general, has been attempted [210]. The latter study noted a reduction of serum IL-6, reductions in craving, cognitive functioning improvements, and reduction in negative psychosocial impacts following administration of *Lachnospiraceae* and *Ruminococcaceae*. The authors also reported an increased abundance of *Roseburia* in FMT-recipients. Interestingly, *Faecalibacterium* and *Roseburia* have been implicated to have a protective role on GBA and intestinal epithelium in alcoholism [211, 212]. Thus, the possibility exists that by restoration of beneficial bacteria significant improvement in CNS health can be achieved. Moreover, manipulation of TLRs as discussed above, could offer additional targets. It is anticipated that with continuous studies in this field, further refinement of treatment modalities involving GM in addiction in general and AUD, in particular may be achieved [207].

## Other therapeutic potentials

In addition to manipulation of GM, extensive effort is being expended in understanding the neurobiological substrates of AUD with the hope of discovering effective novel targets [212]. As it currently stands, three approved medications are available to combat alcoholism or AUD, aiming to stop or reduce the drinking habit and prevent relapse. These include disulfiram, an inhibitor of the degrading enzyme aldehyde dehydrogenase, that acts by inducing aversion nalmefene or naltrexone, antagonists of opioid receptors that act by blunting the rewarding effects of alcohol, and acamprosate, a gamma amino butyric acid (GABA) synthetic analog that acts by modulating or antagonizing NMDA receptors. The latter is primarily used for maintenance of abstinence from alcohol in detoxified alcohol-dependent patients [213]. However, all these medications are only modestly effective. In addition, about one in six people globally, is estimated to receive treatment, with the rate being at even lower in low and lower-middle-income countries [214]. For potentially life-threatening condition, manifested during withdrawal and believed to be caused by glutamate overactivity, benzodiazepine are the primary medications applied [215]. In addition, “talk therapy” or behavioral interventions, consisting of therapies that build motivation and teach skills for coping and preventing relapse, when combined with medications yield a better outcome. Physical activity may also be used as adjunctive treatment for severe AUD [216]. Potential application of neurosteroids, polyphenols, neuropeptides, modulators of nicotinic acetylcholine receptors [217], muscarinic acetylcholine receptors, glutamate receptors, GABA receptors, cannabinoid receptors, G protein-coupled receptors (GPCRs), tyrosine-kinase receptors as well as various nutrients such as carnitine, folic acid, selenium, omega 3 fatty acids and zinc were recently reviewed [212].

## Discussion

Adolescence is a period of human development that span between childhood and adulthood. The neurodevelopmental transformations during adolescence are geared towards acquiring cognitive and social skills that are required to enable the dependent teen to eventually transform to an independent adult. However, some developmental or maturation imbalance in circuitries that control reward vs. inhibition in adolescence, can lead to increased presentation of risk-taking and reward-seeking behaviors, which can include heightened risk of substance abuse such as alcohol drinking. Mirroring the adolescent neurodevelopmental changes, the gut microbiota also undergoes significant maturation, and at the same time establishes a strong bidirectional communication with the brain. This reciprocal communication, referred to as GBA plays a crucial role in driving the behavioral changes associated with AUD.

There are emerging mechanisms by which altered microglial functions could contribute to several major etiological factors of AUD. Pre- and postnatal exposure to alcohol can modulate microglial cell phenotype and function, supporting the notion that reciprocal interactions between microglia and intestinal microbes could play a crucial role in AUD etiology. Alcohol-associated inflammatory signaling contributes not only to CNS inflammation and neurodegeneration but also to alcohol addiction.

**FIGURE 1:**
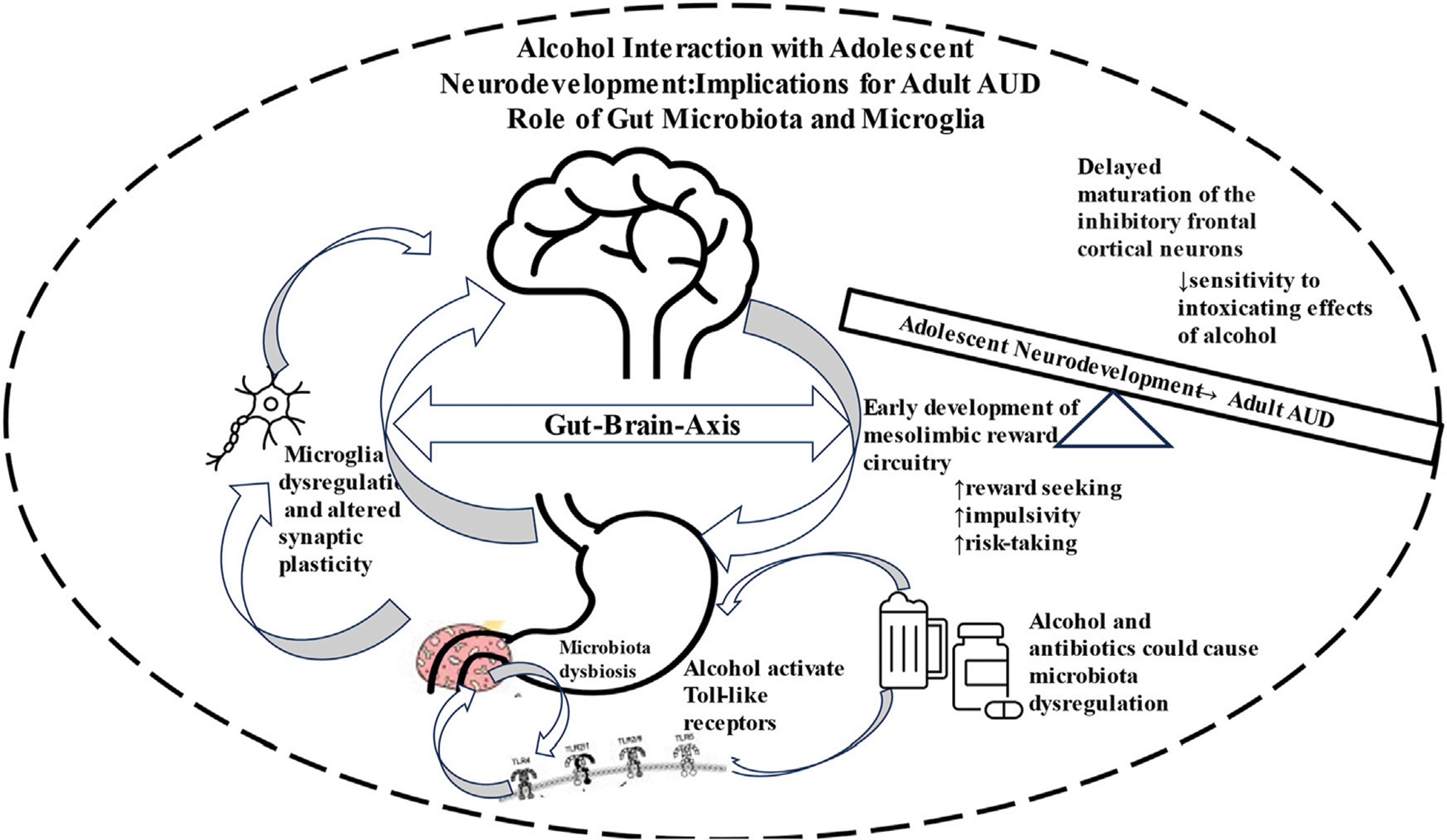
Schematic diagram depicting involvement of Gut-Brain Axis in neurodevelopment that renders the adolescents more vulnerable to drug seeking behavior and eventual manifestation of alcohol use disorder (AUD).

Chronic and high alcohol use can cause GM dysbiosis, leading to neuroinflammatory condition via microglia activation and eventual manifestation of AUD (Figure 1). It is estimated that adolescents who begin drinking alcohol between the ages of 11–14 are 4 times more likely to develop AUD compared to peers that postponed drinking until after the age of 20.

Based on crucial role of GM and microglia in AUD manifestation, particularly during adolescence, and our deeper understanding of the interaction between these two systems, novel promising interventions are presented. However, further investigation on not only the efficacy of the approaches but also the potential role of gender and/or ethnicity in AUD manifestation and treatment are of crucial importance.

## Abbreviations:

ALD: alcoholic liver disease
AUD: Alcohol Use Disorder
AW: alcohol withdrawal
AD: Alzheimer’s Disease
BBB: blood-brain-barrier
BAL: blood alcohol level
CDC: Centers for Disease Control
CNS: central nervous system
DA: dopamine
DR1: Dopamine Receptor D1
FASD: fetal alcohol syndrome disorder
FFAR: free fatty acid receptor
GABA: gamma amino butyric acid
GF: germ-free
GM: Gut microbiota
GBA: Gut-brain-axis
GPCR: G protein-coupled receptors
IL: interleukin
LPS: lipopolysaccharide
NFκB: nuclear factor-κB
NIAAA: National Institute of Alcohol Abuse and Alcoholism
NMDA: N-methyl-D-aspartate
PFCX: prefrontal cortex
SAMHSA: Substance Abuse and Mental Health Services
SCFA: short chain fatty acids
SPF: specific pathogen-free
TBI: traumatic brain injury
TLRs: Toll-like Receptors
